# Pediatric Thrombolysis: A Practical Approach

**DOI:** 10.3389/fped.2017.00260

**Published:** 2017-12-06

**Authors:** Cristina Tarango, Marilyn J. Manco-Johnson

**Affiliations:** ^1^Division of Hematology, Department of Pediatrics, Cancer and Blood Diseases Institute, Cincinnati Children’s Hospital Medical Center Cincinnati, University of Cincinnati, Cincinnati, OH, United States; ^2^Department of Pediatrics, Section of Hematology, Oncology, and Bone Marrow Transplantation, University of Colorado Anschutz Medical Campus, Children’s Hospital, Aurora, CO, United States

**Keywords:** pediatric thrombosis, pediatric thrombolysis, DVT therapy, pediatric DVT, thrombolysis, combination therapy

## Abstract

The incidence of pediatric venous thromboembolic disease is increasing in hospitalized children. While the mainstay of treatment of pediatric thrombosis is anticoagulation, reports on the use of systemic thrombolysis, endovascular thrombolysis, and mechanical thrombectomy have steadily been increasing in this population. Thrombolysis is indicated in the setting of life- or limb-threatening thrombosis. Thrombolysis can rapidly improve venous patency thereby quickly ameliorating acute signs and symptoms of thrombosis and may improve long-term outcomes such as postthrombotic syndrome. Systemic and endovascular thrombolysis can result in an increase in minor bleeding in pediatric patients, compared with anticoagulation alone, and major bleeding events are a continued concern. Also, endovascular treatment is invasive and requires technical expertise by interventional radiology or vascular surgery, and such expertise may be lacking at many pediatric centers. The goal of this mini-review is to summarize the current state of knowledge of thrombolysis/thrombectomy techniques, benefits, and challenges in pediatric thrombosis.

## Introduction

The incidence of pediatric venous thromboembolism (VTE) is estimated to be 0.07–0.14/10,000 children (1, 2), and data suggest that the incidence of thrombosis in children is dramatically increasing (3). This increase may be due in part to the advancement in the management and invasive supportive care of critically ill children and improved imaging modalities to diagnose VTE. Several risk factors for pediatric thrombosis have been identified, including the presence of a central venous catheter (CVC), cancer, congenital heart disease, and surgery (4).

The mainstay of therapy of acute pediatric thrombosis is anticoagulation, and the goals of anticoagulation are to prevent propagation of acute thrombosis, prevent recurrence, and prevent embolization. The most commonly used anticoagulants are unfractionated heparin (UFH), low-molecular-weight heparins (LMWH), and vitamin K antagonists (5). Unfortunately, anticoagulation alone does not rapidly restore the patency of occluded vessels, and in patients at high risk for acute venous insufficiency, VTE recurrence, or postthrombotic syndrome (PTS), anticoagulation may not be enough to achieve optimal outcomes. Moreover, the additional risk factors for and the frequency of adverse outcomes in pediatric thrombosis remain poorly understood. Recurrent thrombosis can occur in 7–8% of children (2, 4). It is estimated that nearly 2% of pediatric venous thrombosis will directly result in death (4), with 4–9% mortality in arterial thrombosis (6). Long-term complications can include limb-length discrepancies and claudication with arterial thrombosis (7), and PTS with venous thrombosis. PTS is the most common chronic complication of VTE and can manifest as pain, edema, and venous stasis ulcers; PTS can limit age-appropriate activities, resulting in a significant impact on children’s quality of life (8). PTS is seen in about 26% of children with extremity venous thrombosis (9).

Thrombolysis has been used in pediatrics for decades (10–15), but in the last 10–20 years, its use has gained momentum with the improved capability of laboratory monitoring, radiologic imaging, interventional radiology, and surgical interventions. In pediatrics, one can consider two broad groups of patients that account for most thrombolysis therapy for VTE. The first group of candidates for thrombolysis may be children and adolescents who present with community(home)-acquired VTE. The second group consists of children with complex congenital heart disease or chronic conditions who develop venous insufficiency related to abnormal hemodynamics, surgical interventions, and life-dependence on CVCs (16, 17). Yet, without high-quality clinical trials of thrombolysis in pediatric thrombosis, providers are left without clear indications for the use of thrombolysis and without uniform dosing regimens. Most importantly, we have limited data on the risks and outcomes of thrombolysis. With this in mind, we will review current data on thrombolysis and offer guidance on its use in pediatric thrombosis outside of the central nervous system.

## Background

### Fibrinolytic System

The fibrinolytic system is a dynamic system that continues to develop following birth (14), and is regulated by various cofactors and inhibitors (18). During fibrinolysis, the zymogen plasminogen is activated by two main serine proteases, tissue-type plasminogen activator (tPA) and urokinase-type plasminogen activator (uPA). tPA binds to fibrin at lysine binding sites and converts plasminogen into plasmin. uPA has no fibrin specificity and can activate both fibrin-bound and circulating plasminogen (14). Both activators have a short half-life of 4–8 min due to the actions of circulating inhibitors: plasminogen activator inhibitor (PAI-1), thrombin-activatable fibrinolysis inhibitor (TAFI), and alpha-2 antiplasmin, which inactivate plasmin or impair plasmin formation (18).

Neonates and infants have qualitative and quantitative differences in plasminogen compared with older children and adults (19). Plasminogen levels increases to adult levels by 6 months of age (20). TPA is also decreased at birth while PAI-1 levels are normal or increased; levels of tPA and PAI-1 do not reach adult levels until late adolescence. Thus, fibrinolysis throughout childhood may be downregulated. Low levels of plasminogen have been shown to impact the actions of pharmacologic thrombolytics (21), but there are insufficient data to demonstrate what effect, if any, high levels of PAI-1 or other fibrinolysis inhibitors may have on the activity of thrombolytic therapy throughout childhood.

## Pharmacologic Agents

In contrast to anticoagulants that decrease the body’s ability to form new thrombus, thrombolytic agents act by converting plasminogen to plasmin and thereby actively reduce clot burden. Several thrombolytics have been used in pediatrics: streptokinase, urokinase, and recombinant tPA (rtPA). In an *in vitro* model of thrombolysis using the three agents, streptokinase showed the slowest rate for clot lysis, tPA had improved lysis early on, and urokinase showed better fibrinolytic specificity (22). Recombinant tPA has a high affinity for fibrin, and the fibrin-tPA complex enhances the binding of plasminogen to fibrin, localizing the effects to the site of thrombosis. rtPA is recommended in pediatrics over other thrombolytics (23), and our review will focus on rtPA.

Recombinant tPA was first FDA approved in the 1980s (24) and initially was used in adults for coronary artery thrombolysis and has since been widely used for stroke (25) and unstable pulmonary embolism (26). The earliest reports in pediatrics were the use of systemic rtPA for catheter-associated arterial thrombosis (11, 13) and pulmonary embolism (12). There are several formulations of rtPA: alteplase with a half-life of 3–5 min, and two modified rtPAs: reteplase^®^ with a half-life of 13–16 min, and tenecteplase with a half-life of 20–24 min. Alteplase is most commonly used in pediatrics due to its short half-life, and dosing for thrombolysis in children is not standardized.

## General Considerations

### Recommended Resources

To improve the safety of and optimize outcomes in patients receiving thrombolysis, a multidisciplinary approach is needed (27). The ability to quickly obtain coagulation testing results for ongoing adjustments in therapy is critical for managing patients receiving thrombolysis and concomitant anticoagulation. Thrombolysis should occur in the critical care setting to allow for rapid intervention should bleeding occur. Access to imaging modalities such as duplex ultrasound, computed tomography, and magnetic resonance imaging also allows for the necessary surveillance of thrombolysis. For endovascular thrombolysis, experienced interventional radiologists or interventional cardiologists familiar with techniques in young patients must be available.

### Laboratory Monitoring

Whether systemic or endovascular thrombolysis is used, concomitant use of anticoagulation is recommended to prevent new thrombus formation during thrombolysis, as clot lysis releases active thrombin which was bound to thrombi (28). Reported dosing of concomitant anticoagulation has ranged from therapeutic UFH to UFH at a set dose of 5–10 units/kg/h (29–31). While UFH therapy alone can be monitored using either aPTT or anti-Xa levels, anti-Xa levels should be monitored during thrombolysis when possible. Fibrin split products can prolong the activated thromboplastin time (aPTT), thus aiming for a specific aPTT is of unclear utility during thrombolysis. Infants or any child with suspected acquired plasminogen deficiency should receive fresh frozen plasma prior to initiation of thrombolysis. Careful laboratory monitoring during thrombolysis is required, with hemoglobin/hematocrit, platelet count, fibrinogen, fibrin degradation products, d-dimer, aPTT, prothrombin time, and UFH anti-Xa levels done every 6–12 h. d-Dimer levels can help direct systemic thrombolysis therapy, as a normal or low d-dimer indicates a lack of thrombolysis and can be used to guide dose increases, while an elevated d-dimer indicates that chemical activation of fibrinolysis has been achieved. A current blood type and screen is also recommended for any patient receiving thrombolysis, as is a renal panel for patients requiring contrast for venography or undergoing mechanical thrombolysis, due to a risk of hemolysis with the latter.

### Timing of Thrombolysis

In general, thrombolysis is used in acute thrombosis of less than 14 days duration of vessel occlusion. In one study assessing efficacy of systemic thrombolysis, 83% of patients with thrombus less than 2 weeks had full or partial response to rtPA compared with 25% in those patients where the thrombus was older (32). For endovascular pharmacomechanical thrombolysis, though, some investigators suggest that more than 60 days from the onset of symptoms is a contraindication (33), although recent attempts to revascularize chronic venous occlusions are proving promising and can be considered for high-risk thrombi (34, 35).

### Precautions and Contraindications

Several precautions should be taken during thrombolysis:

- No arterial punctures or line placements.
- No intramuscular injections.
- Minimal manipulation of the patient (e.g., no chest physiotherapy).
- No urinary catheterization, rectal temperatures, nasogastric tube placement.
- Blood samples should be obtained from a superficial vein or indwelling catheter.
- Avoid concurrent NSAIDs or anti-platelet therapy.
- Intracranial imaging should be considered prior to and after thrombolytic therapy in children less than 3 months of age or any child at high risk for ischemic or hemorrhagic stroke.

While the decision to use thrombolysis should be made on a case-by-case basis, weighing pertinent risks, and benefits, there are general contraindications to thrombolysis that should be considered (27, 36).

- Active bleeding.
- Concurrent bleeding diathesis: inability to maintain platelets greater than 100,000/μl and fibrinogen above 100 mg/dL using transfusion support.
- Recent major surgery or trauma, puncture of a noncompressible vessel, or organ biopsy within the previous 10 days.
- Intracranial hemorrhage, infarction or intracranial or spinal surgery within the last 2 months.
- Known right-to-left intracardiac shunt.
- Cardiopulmonary resuscitation or asphyxia within 7 days of therapy (including complicated birth).
- Extreme prematurity.

Other contraindications to thrombolysis have included pregnancy or puerperium; presence of intracranial vascular malformations, aneurysms or neoplasms; uncontrolled hypertension; infective endocarditis; and any contraindication to the use of unfractionated heparin or radiographic contrast media (if needed for assessment of thrombosis) (27).

## Indications and Patient Selection

Acute or subacute occlusive venous or arterial thrombosis that is limb- or life-threatening is the primary indication for thrombolysis. Thrombolysis can improve limb and organ perfusion by improving vessel patency and may quickly improve symptoms. Consensus guidelines offer indications for thrombolysis (23), but its use must be carefully considered due to the potential higher risk of major bleeding compared with anticoagulation alone.

### Strong Indications for Thrombolysis

- Arterial thrombosis with tissue ischemia.
- Phlegmasia alba/cerulea dolens: extensive venous thrombosis with total occlusion of venous flow, increased compartment pressures and compromise of arterial blood flow (37).
- Pulmonary embolism (PE) with hypotension or shock, or PE resulting in right heart strain or myocardial necrosis.
- Superior vena cava syndrome.
- Bilateral renal vein thrombosis.
- Congenital heart disease with shunt thrombosis.
- Large (>2 cm), mobile right atrial thrombus.
- Kawasaki disease with coronary artery thrombosis.
- Cerebral sinovenous thrombosis with neurologic impairment and no improvement with anticoagulation or progressive thrombosis.

### Possible Indications for Thrombolysis

In the past decade, there have been reports of the use of thrombolysis in children for indications beyond acute limb- or life-threatening situations (31, 33, 38, 39). The goal of therapy in these cases is often to improve long-term outcomes or to maintain venous patency in children dependent on central venous access (17). Small prospective and retrospective case series suggest that the use of thrombolysis may be indicated for occlusive, symptomatic iliofemoral or inferior vena cava DVT to acutely decrease pain and improve function and, long-term, to potentially decrease the risk of (PTS) (31, 33, 38). Thrombolysis has been shown to decrease PTS in adults (40). Venous compression syndromes such as May-Thurner (41, 42) and Paget Schroetter (43) (effort thrombosis) have also been treated with combined thrombolysis and endovascular or surgical techniques. Table 1 shows the results of published case series or cohort studies and demonstrates convergent results in thrombolysis efficacy, and rates of recurrence, PTS, and major bleeding.

**Table 1:** Published results of thrombolysis in children.

Author	Method	*N*	Age, range and site of thrombosis	Lysis^a^	Major hemorrhage	SAEs, other	Recurrent VTE	PTS
Manco-Johnson (28)	Systemic UK/UH	32	6 weeks to 17 years and UE, LE, SVC, IVC, PE, atrial	50%	0	Death 1; PE 1; progress 1	9%	11.1% MJ

Wang (29)	Systemic TPA	12 HD 17 LD	1 day to 17 years and LE, UE, PE, CSVT, renal, hepatic, arterial, and venous	92% 100%	0 1 ICH, PT infant	1 embolic stroke with left atrial thrombus	0	8% 0% MJ

Goldenberg (38)	Systemic/PPMT	9	1–21 years and LE	89%	1 pulmonary	0	0%	11.1% MJ

Goldenberg (33)	CDT/PMT/PPMT	16	11–19 years and LE and UE	88%	0	PE 1	27%	13% MJ

Darbari (39)	CDT/PMT/PPMT	34	13 days to 21 years and LE and UE	17%(52%) 50 (99%)	1 2 required prbcs	0%	NA	NA

Dandoy (31)	CDT/PMT/PPMT	41	3 months to 21 years and LE, UE, SVC, and IVC	90% (>50%)	1 Required prbcs	PE 1	NA	14% (V or mV)

Gaballah (55)	CDT/PMT/PPMT	57	1–17 years and LE	33% (93.7%) >50%	1.8%		12%	2.1%V 59.3% mV

*^a^Lysis indicates 90–100% clot lysis; studies reports indicating 50–99% lysis or >50–% lysis are so noted*.

*N, number of cases; SAE, serious adverse events; VTE, venous thromboemobolism; PTS, postthrombotic syndrome; UK, urokinase; UH, unfractionated heparin; TPA, tissue plasminogen activator; ICH, intracranial hemorrhage; PT, preterm; PE, pulmonary embolism; MJ, Manco-Johnson PTS scale; PPMT, percutaneous pharmacomechanical thrombolysis; CDT, catheter-directed thrombolysis; PMT, percutaneous mechanical thrombolysis; prcs, packed red cells; NA, not available; V, Villalta PTS scale; mV, modified Villalta PTS scale. LE, lower extremity; UE, upper extremity; SVC, superior vena cava; IVC inferior vena cava*.

## Methods of Thrombolysis

The choice of either systemic or endovascular thrombolysis depends on several factors. Foremost, is the availability of interventional radiologists/cardiologists who have pediatric expertise with delivering site-directed endovascular treatment. There are sparse data on endovascular thrombolysis in certain anatomic areas such as the pulmonary vasculature (45), or cerebral venous sinuses (46), so systemic thrombolysis, rather than endovascular, is recommended for these sites. Another factor that may impact the mode of thrombolysis is the acuity of impending loss of life or limb, as the time to coordinate endovascular thrombolysis may be prohibitive. Also, a patient at higher risk of bleeding may benefit from endovascular thrombolysis as some data suggests that endovascular thrombolysis has a lower risk of bleeding than systemic thrombolysis (47).

### Systemic Thrombolysis

Systemic thrombolysis is the intravenous administration of thrombolytics distant from the site of thrombosis. Case series using rtPA in children have used various dosing regimens with differing rates of success (29, 48, 49). A low-dose rtPA infusion and a high-dose (previously referred to as “standard dose”) rtPA regimen have been described, with low-dose therapy showing equivalent efficacy to a high-dose regimens (29, 30). High-dose rtPA can be used for 6 h at a time and may be repeated over a 72-h period if imaging suggests no response. Low-dose rtPA can be a continuous infusion over 6–72 h, with close laboratory monitoring and imaging at a minimum of daily. Table 2 describes dosing regimens.

**Table 2:** Dosing of alteplase and heparin during thrombolysis.

Mode of thrombolysis	Alteplase dosing		Duration of thrombolysis	Concomitant UFH therapy	Laboratory monitoring
	Bolus	Infusion			
Systemic thrombolysis	None None	Low-dose: 0.01–0.06 mg/kg/h (max 2 mg/h) High-dose: 0.1–0.5	6–72 h 2–6 h, may repeat if indicated	Prophylactic UFH with goal UFH anti-Xa level of 0.1–0.3 *or* UFH at 10 U/kg/h	Every 6–12 h: fibrinogen, CBC, FDPs, PT, aPTT, UFH anti-Xa

Site-directed thrombolysis	0.1–0.3 mg/kg (max dose 10 mg)	0.01–0.03 mg/kg/h *or* max 1–2 mg/h	Up to 72––96 h	Therapeutic UFH with goal UFH anti-Xa level of 0.3–0.7 *or* ufh at 10 U/kg/h	Every 6–12 h: fibrinogen, CBC, FDPs, PT, aPTT, UFH anti-Xa, renal profile, urinalysis

In a review of pediatric thrombolysis, the reported overall number of patients with complete or partial resolution of thrombosis with systemic thrombolysis was 79%, with an incidence of major bleeding in 15% (47). Major bleeding is most often defined as fatal bleeding, bleeding resulting in a drop in hemoglobin of 2 g/dL or more within 24 h, retroperitoneal, pulmonary or intracranial, and bleeding requiring surgical intervention (50). Major bleeding events in children receiving systemic thrombolysis are often associated with longer tPA infusion times and a lower fibrinogen level immediately after thrombolysis (48).

### Endovascular Thrombolysis

No studies have compared endovascular to systemic thrombolysis in children. Advantages of directed therapy are lower doses of rtPA, with potentially less risk of bleeding, and the ability to instill the thrombolytic agent directly into or near the thrombus. Endovascular thrombolysis is invasive, and is more costly than systemic thrombolysis due to the need for pediatric intensive care for up to 4 days, requirement of general anesthesia, use of interventional radiology expertise and suite for up to three sessions. In a systematic review of pediatric thrombosis, endovascular thrombolysis resulted in complete resolution in 76%, partial resolution in 17%, and no resolution in 7% of cases (47). Endovascular thrombolysis can be done over 12–96 h and can include several interventions (51):

- Infusion-only, catheter-directed thrombolysis (CDT): This is the placement of a catheter under radiologic imaging directly into the thrombus to deliver thrombolytic agent.
- Percutaneous mechanical thrombolysis/thrombectomy (PMT): This is the use of intravascular aspirating-type devices without thrombolytic to mechanically remove thrombus. This form of thrombolysis is discouraged because of the possibility of vascular injury and is used when thrombolytics are contraindicated.
- Percutaneous pharmacomechanical thrombolysis (PPMT): This is the combined use of CDT with a device that mechanically breaks up the thrombus. A commonly used device in children is the Angiojet™, which uses high-velocity saline jets to generate strong negative pressures to break up and suction out the thrombus. The availability of Angiojet 4Fr devices allows for use in small vessels. The Ekos™ system uses ultrasound to instill tPA into the clot and is also gaining acceptance. These devices may cause significant hemolysis (52); intravenous hydration and monitoring of serial creatinine/renal function and electrolytes is recommended.

There is no standard protocol or preferred device in pediatrics, and use of any of these modalities is often physician-dependent. By allowing direct access to the occluded vessel, endovascular therapy can allow for additional techniques such as angioplasty and stent placement to improve vessel patency. Retrievable IVC filters may be placed at the time of endovascular thrombolysis to prevent embolization (53) but are not always necessary and are placed at the discretion of the interventionalist. If placed, IVC filters can be removed at the end of the procedure or within 3 months of placement. Pulmonary embolism is a known complication of endovascular thrombolysis and has been reported in 1–3% (44, 54). Major bleeding in children undergoing endovascular thrombolysis has been reported to be 0–3% (31, 33, 39, 55). Surgical thrombectomy has been reported in severe pediatric thrombosis where anticoagulation and thrombolysis have failed (56), cardiac ventricular thrombosis (57), and IVC thrombosis associated with abdominal tumors (58). Surgical thrombectomy should be reserved for the direst cases.

## Management of Bleeding Complications

Bleeding is the most feared complication of thrombolytic therapy. Transfusion of cryoprecipitate should be given for hypofibrinogenemia (<100 mg/dL) and the platelet count should be maintained above 100 K/μL during thrombolysis. Menstruating females may receive non-estrogen-containing hormonal suppression with norethindrone before or during thrombolysis. Minor bleeding, such as bleeding from intravenous lines or catheterization site, can be managed with local control (pressure bandages or topical hemostatic agents, e.g., topical thrombin.) If more extensive bleeding occurs, the rtPA infusion can be decreased or temporarily stopped for at least 1 h. Anticoagulation can also be held or the dose lowered if bleeding persists. When bleeding is controlled, the anticoagulation can then be started at lower dose if previously stopped, and the rtPA infusion can be restarted at a lower dose. For major bleeding, such as intracranial or intra-abdominal bleeding, anticoagulation and rtPA should be stopped and cryoprecipitate should be administered. UFH can be reversed with protamine (1 mg of protamine for 100 U of heparin, maximum protamine dose is 50 mg/dose), and an antifibrinolytic such as aminocaproic acid or tranexamic acid be administered (23), although antifibrinolytics are not standard in this setting. Emergent surgical intervention may be required for major bleeding.

## Summary and Conclusion

As the incidence of thrombosis in children increases, providers must be aware of treatment options to optimize outcomes. PTS occurs in nearly 30% of pediatric patients with thrombosis, which can cause life-long signs and symptoms of limb swelling, pain, and limitations in normal activities. Adult studies suggest that that thrombolysis decreases the risk of PTS by a third (59), and data in pediatrics also suggest that thrombolysis can significantly decrease the incidence of this complication (38). As children are expected to live for decades after a DVT, providers should strongly consider treatment modalities that may decrease the risk of this chronic complication. Thrombolysis can be safely performed in children but requires extensive monitoring and collaboration with hematology, critical care, and in cases of endovascular therapy, interventional radiology or interventional cardiology. Until randomized trials are performed to assess the benefits, risks, and complications of thrombolytic therapy in children, clinicians will need to continue careful patient selection and establish short- and long-term monitoring of patients treated with this therapy.

## Author Contributions

CT wrote the manuscript. MM-J reviewed the manuscript.

## Conflict of Interest Statement

The authors declare that the review was conducted in the absence of any commercial or financial relationships that could be construed as a potential conflict of interest.
